# Клинические рекомендации «Острые и хронические тиреоидиты (исключая аутоиммунный тиреоидит)»

**DOI:** 10.14341/probl12747

**Published:** 2021-04-12

**Authors:** Е. А. Трошина, Е. А. Панфилова, М. С. Михина, И. В. Ким, Е. С. Сенюшкина, А. А. Глибка, Б. М. Шифман, А. А. Ларина, М. С. Шеремета, М. В. Дегтярев, П. О. Румянцев, Н. С. Кузнецов, Г. А. Мельниченко, И. И. Дедов

**Affiliations:** Национальный медицинский исследовательский центр эндокринологии; Национальный медицинский исследовательский центр эндокринологии; Национальный медицинский исследовательский центр эндокринологии; Национальный медицинский исследовательский центр эндокринологии; Национальный медицинский исследовательский центр эндокринологии; Национальный медицинский исследовательский центр эндокринологии; Национальный медицинский исследовательский центр эндокринологии; Национальный медицинский исследовательский центр эндокринологии; Национальный медицинский исследовательский центр эндокринологии; Национальный медицинский исследовательский центр эндокринологии; Национальный медицинский исследовательский центр эндокринологии; Национальный медицинский исследовательский центр эндокринологии; Национальный медицинский исследовательский центр эндокринологии; Национальный медицинский исследовательский центр эндокринологии

**Keywords:** клинические рекомендации, хронический тиреоидит, подострый тиреоидит, острый тиреоидит, амиодарониндуцированный тиреоидит, тиреоидит Риделя, цитокининдуцированный тиреоидит, тиреотоксикоз, гипотиреоз

## Abstract

Острые и хронические заболевания щитовидной железы занимают второе место по выявляемости после сахарного диабета. Всемирная организация здравоохранения отмечает ежегодную тенденцию к увеличению числа заболеваний щитовидной железы. В настоящих клинических рекомендациях будут рассмотрены вопросы этиологии, клинического течения, диагностики и лечения острых и хронических (за исключением аутоиммунного) воспалительных заболеваний щитовидной железы.Клинические рекомендации — это основной рабочий инструмент практикующего врача, как специалиста, так и врача узкой практики. Лаконичность, структурированность сведений об определенной нозологии, методов ее диагностики и лечения, базирующихся на принципах доказательной медицины, позволяют в короткий срок дать тот или иной ответ на интересующий вопрос специалисту, добиваться максимальной эффективности и персонализации лечения.Клинические рекомендации составлены профессиональным сообществом узких специалистов, одобрены экспертным советом Министерства здравоохранения РФ. Представленные рекомендации содержат максимально полную информацию, которая требуется на этапе диагностики острых и хронических тиреоидитов, этапе выбора тактики ведения пациентов с тиреоидитом, а также на этапе лечения пациента.Рабочая группа представляет этот проект в профессиональном журнале, посвященном актуальным проблемам эндокринологии, с целью повышения качества оказываемой медицинской помощи, повышения эффективности лечения острых и хронических тиреоидитов путем ознакомления с полным тестом клинических рекомендаций по острым и хроническим тиреоидитам (исключая аутоиммунный тиреоидит) максимально возможного количества специалистов в области не только эндокринологии, но и медицины общей (семейной) практики.

## Abstract (en)

Acute and chronic thyroid diseases are the most frequently detected disorders being second only to diabetes mellitus.The World Health Organization points out that thyroid diseases’ incidence tends to grow every year. The present paper consists of clinical practice guidelines that consider etiology, clinical course, diagnostics and treatment of acute and chronic inflammatory thyroid diseases (except those of autoimmune type).The clinical practice guidelines provide an important working tool for clinicians including specialty physicians and medical experts. Containing structured and concise information on the specific nosology, diagnostic methods and treatment tips these guidelines allow medical specialists to quickly resolve difficulties and choose the most efficient and personalized treatment (following strict principles of evidence-based medicine at the same time).The clinical practice guidelines were drawn up by highly-skilled professional team of specialty physicians approved by the Expert Council of Russian Federation’s Health Department. These guidelines contain the most complete and up-to-date information required to diagnose acute and chronic thyroiditis, provide patient care and treatment.The working group publishes the present paper in the professional journal dealing with endocrinology topics to improve healthcare quality and refine treatment of acute and chronic thyroiditis (autoimmune thyroiditis excluded). It is advisable to acquaint as many endocrinology and general (family) medicine specialists as possible with the full text of these clinical guidelines.

## СПИСОК СОКРАЩЕНИЙ

## ТЕРМИНЫ И ОПРЕДЕЛЕНИЯ

Компрессионный синдром — совокупность симптомов сдавления окружающих органов и тканей. Компрессионный синдром вследствие значительного увеличения щитовидной железы протекает с нарушением глотания и дыхания, приводящих к развитию механической асфиксии и дыхательной недостаточности.

Мультифокальное фиброзирующее расстройство — прогрессирующее развитие плотной фиброзной соединительной ткани неизвестной этиологии, захватывающее многие органы и ткани.

## 1.КРАТКАЯ ИНФОРМАЦИЯ ПО ЗАБОЛЕВАНИЮ ИЛИ СОСТОЯНИЮ (ГРУППЕ ЗАБОЛЕВАНИЙ ИЛИ СОСТОЯНИЙ)

## 1.1. ОПРЕДЕЛЕНИЕ ЗАБОЛЕВАНИЯ ИЛИ СОСТОЯНИЯ (ГРУППЫ ЗАБОЛЕВАНИЙ)

Тиреоидиты — это группа заболеваний щитовидной железы (ЩЖ), различных по этиологии, патогенезу, клиническим проявлениям [1].

В связи с тем, что эта группа заболеваний чрезвычайно гетерогенна, для удобства прочтения авторский коллектив вынужден был внести незначительные изменения в структуру клинических рекомендаций, представив вначале классификацию, а затем описав каждую нозологическую форму в отдельности.

Аутоиммунный тиреоидит (АИТ) в настоящих клинических рекомендациях не рассматривается. Безболевой (молчащий), а также послеродовый тиреоидит как варианты аутоиммунного тиреоидита в настоящих клинических рекомендациях не описаны [2].

Острый тиреоидит (ОТ, острый гнойный тиреоидит, бактериальный тиреоидит, острый струмит) — острое воспаление ЩЖ, вызванное бактериальной инфекцией.

Острый негнойный тиреоидит — воспалительное заболевание ЩЖ в результате лучевого воздействия (радиойодтерапии (РЙТ)), травмы или кровоизлияния в ЩЖ.

Подострый тиреоидит (ПТ; синонимы: гранулематозный тиреоидит, тиреоидит Де Кервена, вирусный тиреоидит, гигантоклеточный тиреоидит) — это заболевание ЩЖ воспалительного характера, предположительно вирусной этиологии, длящееся от одной недели до нескольких месяцев, в разгар заболевания чаще всего проявляющееся выраженной болезненностью в области ЩЖ и лихорадкой, иногда с присоединением симптомов тиреотоксикоза; имеющее склонность к рецидивированию [3–5].

Амиодарониндуцированные тиреоидиты — группа заболеваний, сопровождающихся дисфункцией ЩЖ, возникших в результате применения амиодарона** [6].

Цитокининдуцированные тиреоидиты — заболевания ЩЖ, чаще всего деструктивного характера, возникающие в результате использования препаратов на основе цитокинов.

Тиреоидиты в результате применения средств, содержащих литий, — группа заболеваний, сопровождающихся дисфункцией ЩЖ, возникших в результате применения препаратов лития [7][8].

Тиреоидит Риделя (ТР) — редкое заболевание, характеризующееся обширным фиброзом, часто поражающим, помимо ЩЖ, окружающие структуры [9].

## 1.2. ЭТИОЛОГИЯ И ПАТОГЕНЕЗ ЗАБОЛЕВАНИЯ ИЛИ СОСТОЯНИЯ (ГРУППЫ ЗАБОЛЕВАНИЙ)

Различны в зависимости от заболевания.

Острый тиреоидит. У взрослых заболевание преимущественно вызвано Staphylococcus aureus, Streptococcus hemolytica, Streptococcus pneumoniae или анаэробными стрептококками, которые встречаются более чем у 80% случаев [10].

У детей α- и β-гемолитический стрептококки и разнообразные анаэробы обнаруживаются приблизительно в 70% случаев, тогда как смешанные болезнетворные микроорганизмы выявляются у 50% больных и более [11].

Среди других возбудителей острого тиреоидита описаны Salmonella brandenberg, Salmonella enteritidis, Actinomyces naeslundii, Actinobacillus actinomycetemcomitans, Brucella melitensis, Clostridium septicum, Eikenella corrodens, Enterobacter spp., Escherichia coli, Haemophilus influenzae, Klebsiella spp., Pseudomonas aeruginosa, Serratia marcescens, Acinetobacter baumannii и Staphylococcus non-aureus, также в литературе описаны случаи грибкового, туберкулезного, паразитарного и сифилитического поражения ЩЖ.

Прослеживается отчетливая связь появления ОТ c перенесенным острым инфекционным заболеванием ЛОР-органов (тонзиллиты, синуситы, отиты) и пневмониями, преимущественно у пациентов с ослабленной иммунной системой (пациенты с ВИЧ, лимфомой Ходжкина, после аутотрансплантации органов, после химиотерапии, злоупотребляющие алкоголем и др.) [12–14]. Инфицирование происходит гематогенным или лимфогенным путем либо в результате прямого попадания инфекции в ЩЖ при травме и ранении, а также в результате инвазивных медицинских манипуляций (пункционная биопсия, склеротерапия этанолом, лазерная фотокоагуляция, радиочастотная термоаблация). Преимущественно поражается одна доля ЩЖ. Воспалительный процесс в ЩЖ проходит все стадии: альтерации, экссудации, пролиферации.

Частой причиной рецидивирующих тиреоидитов (чаще встречается в детском возрасте) является наличие сообщения (фистулы) доли ЩЖ с грушевидным синусом. Отличительной особенностью этой детской патологии является поражение только левой доли ЩЖ [10][14].

Подострый тиреоидит. Обычно ПТ развивается после перенесенной вирусной инфекции, чаще всего вирусной инфекции верхних дыхательных путей [8]. В пользу вирусной этиологии свидетельствуют длительный продромальный период, эпидемический характер заболеваемости, сезонное (зимой и осенью) увеличение случаев заболеваемости. Предполагаемые возбудители: вирус эпидемического паротита, вирус Коксаки, аденовирусы, ЕСНО-вирусы, вирусы гриппа, в том числе H1N1, вирус Эпштейна–Барр. Вирус эпидемического паротита культивируется непосредственно из ткани ЩЖ, вызванной подострым тиреоидитом, по-видимому, являясь особым этиологическим фактором. Кроме того, ПТ был ассоциирован с другими вирусными состояниями, такими как инфекционный мононуклеоз, ВИЧ и др. [15–19].

Существует генетическая предрасположенность к ПТ, так как заболеваемость выше у лиц с HLA-BW35 [4].

В одной из работ риск развития рецидива ПТ был HLA-зависимым, а определяющим фактором было совместное присутствие HLA-B*18:01 и -B*35. Авторы полагают, что у таких пациентов необходим режим лечения с применением высоких доз глюкокортикостероидов и медленное их снижение [20].

В другом исследовании с участием 60 пациентов с ПТ и 1023 здоровых лиц из группы контроля, в дополнение к ранее описанной связи ПТ с HLA-B*35, генетическая восприимчивость к ПТ была связана с наличием HLA-B *18: 01, DRB1*01 и C*04:01, аллели HLA-B*18:01 и DRB1*01 были независимыми факторами риска ПТ [21].

Проникая внутрь клетки, вирус вызывает образование атипичных белков, на которые организм реагирует воспалительной реакцией. Воспалительный процесс в ЩЖ приводит к деструкции фолликулярных клеток и фолликулов, потере фолликулами коллоида. Отмечается инвазия ЩЖ полинуклеарными лейкоцитами, лимфоцитами, образуются гранулемы, которые содержат гигантские многоядерные клетки. Наряду с деструктивными изменениями наблюдаются пролиферация тиреоидных клеток и образование новых фолликулов. Все содержимое поврежденного фолликула железы попадает в кровеносное русло (тиреотоксикоз без гиперфункции ЩЖ) [22][23].

Амиодарониндуцированные тиреоидиты

Амиодарон** — антиаритмический препарат III класса, применяемый для купирования угрожающих жизни суправентрикулярных и желудочковых аритмий, с высоким содержанием йода: 1 таблетка (200 мг) препарата содержит 74 мг йода, при метаболизме которого высвобождается около 7 мг йода в сутки, что во много раз превышает суточную потребность в элементе. Амиодарон** и его метаболит дизэтиламиодарон обладают способностью активно накапливаться некоторыми тканями организма (жировой тканью, печенью, легкими, в меньшей степени скелетной мускулатурой, почками, сердцем, мозгом), а период его полужизни составляет 22–100 дней. Таким образом, амиодарон** и йодированные продукты его метаболизма могут сохраняться в организме долгое время после отмены препарата.

У большинства пациентов, принимающих амиодарон**, отмечаются незначительные изменения уровня гормонов ЩЖ, объясняемые его влиянием на их синтез, транспортировку и высвобождение. Среди основных механизмов влияния амиодарона** на ЩЖ и тиреоидный статус можно отметить следующие.

Тем не менее все вышеописанные процессы, как правило, протекают умеренно с изменяющейся интенсивностью на различных сроках приема амиодарона** и в результате «адаптации» к ним организма в норме не приводят к значимому отклонению тиреоидных гормонов. Однако у отдельных пациентов эти механизмы становятся частью патогенеза более глубоких нарушений — индуцированных амиодароном** тиреопатий [6, 24–26].

Амиодарониндуцированный гипотиреоз

Амиодарониндуцированный тиреотоксикоз

В основе патогенеза тиреотоксикоза на фоне приема амиодарона** лежат два основных механизма, согласно которым выделяют два типа амиодарониндуцированного тиреотоксикоза.

Цитокининдуцированные тиреоидиты

Нередко для лечения онкологических (карциноидные опухоли и др.), вирусных (гепатит В и С и др.) и аутоиммунных заболеваний (рассеянный склероз и др.) используются препараты из группы цитокинов, которые модулируют иммунный ответ.

Цитокины представляют собой небольшие растворимые белки, секретируемые клетками иммунной системы и другими клетками, являются частью межклеточной системы связи, отвечающей за иммунный ответ. Эти белки, связывая специфические клеточные рецепторы, либо индуцируют, либо ингибируют регулируемые ими гены. Во время вирусной инфекции различные цитокины играют роль как в вирусном клиренсе, так и в повреждении тканей [27].

При наличии вируса гепатита С в организме вирион может инфицировать фолликулярные клетки у иммунокомпетентных пациентов, что, вероятно, является потенциальным механизмом развития дисфункции [28].

Однако если вирион не инфицирует клетки ЩЖ, вирусные белки, выделяемые из вирионов, также могут приводить к значимым физиологическим последствиям. Например, было показано, что белки Е2 могут индуцировать апоптоз [29][30] и активировать провоспалительный цитокин-интерлейкин 8 [31].

Тиреоидиты, возникшие в результате применения средств, содержащих литий

Пациенты, принимающие препараты лития для лечения биполярного расстройства, также имеют повышенный риск развития дисфункции ЩЖ: диффузного и узлового зоба, а по некоторым данным, и дисфункции ЩЖ: гипотиреоза и реже тиреотоксикоза [7][8].

Развитие гипотиреоза объясняют несколькими механизмами: прямым ингибированием фермента, ответственного за регуляцию пролиферации тиреоцитов, нарушением захвата йода и высвобождения тиреоидных гормонов, а также снижением чувствительности рецепторов к ТТГ. Из-за выявленной способности лития угнетать функциональную активность тиреоцитов ранее его применение рассматривалось в качестве одной из возможных терапевтических мер при тиреотоксикозе. Таким образом, снижение функции ЩЖ на фоне его приема можно считать закономерным. Развитие зоба объясняется, скорее, не влиянием повышения уровня ТТГ по обратной связи, а непосредственным влиянием на внутриклеточные механизмы пролиферации. Данный вывод следует из результатов исследования, подтвердившего большую частоту развития зоба на фоне терапии литием, а также отсутствие связи между структурными изменениями ЩЖ и уровнем тиреоидных гормонов [32][33].

Кроме того, отмечено, что литий может интенсифицировать развитие имеющего место тиреоидита, увеличивая титр циркулирующих АТ-ТПО. Вместе с тем доказательств способности лития индуцировать синтез АТ-ТПО de novo получено не было. Кроме того, не было выявлено связи между приемом лития и уровнем АТ-рТТГ [34][35].

Одна из форм тиреоидита, связанного с приемом лития, характеризуется преходящим трехфазным течением с возможным исходом в гипотиреоз, аналогичным таковому при безболевом тиреоидите. Однако результаты гистологических исследований пациентов с литий-индуцированным «молчащим» тиреоидитом представляют картину, отличную от безболевого, выявляя разрушения клеток в отсутствие лимфоцитарной инфильтрации [36].

Отсутствие подтверждений связи между приемом лития и уровнем АТ-рТТГ вместе с результатами гистологических исследований свидетельствуют скорее в пользу непосредственного цитотоксического воздействия лития, нежели об аутоиммунном механизме повреждений. Разрушение клеток с высвобождением тиреоидных гормонов рассматривается как наиболее вероятная причина транзиторного тиреотоксикоза при приеме лития. Тем не менее клинически они неотличимы [37].

Тиреоидит Риделя

Этиология ТР неизвестна. Данное заболевание может быть очень редким вариантом хронического аутоиммунного тиреоидита Хашимото или проявлением системного фиброза, связанного с продукцией IgG4. Наличие антител к ткани ЩЖ, эозинофильная инфильтрация и положительный эффект лечения глюкокортикостероидами позволяют предположить аутоиммунный характер поражения. Антитела к тиреопероксидазе (АТ-ТПО) выявляются примерно у 90% пациентов с АИТ.

ТР может сочетаться с фиброзом орбит, первичным склерозирующим холангитом, фиброзом средостения и забрюшинного пространства, являться частью мультифокального идиопатического фиброзирующего расстройства [9][38][39].

Характерной особенностью ТР является замена ткани ЩЖ плотной фиброзной соединительной тканью. Фиброз может распространяться на соседние ткани: околощитовидные железы, мышцы шеи, гортанные нервы, кровеносные сосуды. Фиброзная ткань проникает в скелетные мышцы шеи, плотно спаивается с пищеводом и трахеей, вызывая их сужение. Заболевание имеет злокачественное течение. Агрессивный рост фиброзной ткани продолжается и после удаления зоба, прогрессирует и после повторных операций, направленных на освобождение трахеи от стенозирующих разрастаний грубой фиброзной соединительной ткани [40].

## 1.3. ЭПИДЕМИОЛОГИЯ ЗАБОЛЕВАНИЯ ИЛИ СОСТОЯНИЯ (ГРУППЫ ЗАБОЛЕВАНИЙ)

Приведена для каждого заболевания.

Острый тиреоидит — редкое заболевание. В структуре всей патологии щитовидной железы распространенность составляет 0,1–0,7% [11][41]. При этом у детей заболевание встречается в 92% случаев, остальные 8% — у взрослых, чаще всего в возрасте 20–40 лет [42][43]. У мужчин и женщин заболевание встречается с равной вероятностью.

ПТ является относительно редким заболеванием с частотой выявления 4,9 случая на 100 000 населения в год, причем частота возникновения у женщин преобладает [4]. Хотя болезнь описана во всех возрастах, она редко встречается у детей [44][45]. Чаще страдают лица среднего возраста, однако встречаются случаи заболеваемости и у пожилых [46][47].

Доля ПТ в структуре заболеваний ЩЖ составляет 1–5%. Отмечается увеличение частоты заболеваемости в осенне-зимний период во время эпидемий вирусных заболеваний [4][48].

Степень риска нарушения функции ЩЖ зависит не от ежедневной или накопившейся дозы амиодарона**, а от йодного обеспечения в регионе проживания. У лиц, проживающих в областях с достаточным потреблением йода, чаще развивается амиодарониндуцированный гипотиреоз, а в регионах с низким потреблением йода чаще отмечают тиреотоксикоз.

Распространенность гипотиреоза на фоне приема амиодарона** колеблется от 6% (в странах с низким потреблением йода) до 13% (в странах без йододефицита). Чаще всего амиодарониндуцированный гипотиреоз отмечают у женщин пожилого возраста. Риск развития гипотиреоза у женщин с повышенным содержанием антител к тиреоидной пероксидазе и/или антител к тиреоглобулину возрастает в 13 раз по сравнению с мужчинами.

Амиодарониндуцированный тиреотоксикоз возникает у 0,9–10% пациентов, получающих терапию. Частота развития амиодарониндуцированного тиреотоксикоза зависит от йодного обеспечения: заболевание преобладает в йододефицитных регионах [6][24][26][49].

Цитокининдуцированный тиреоидит

Проведение терапии интерфероном-α повышает риск формирования дисфункции ЩЖ: у 5–10% пациентов манифестируют тиреопатии (первичный гипотиреоз в исходе АИТ, болезнь Грейвса и деструктивный тиреоидит). Чаще развивается гипотиреоз — 3,8%, реже тиреотоксикоз — 2,8%. Редко встречается эндокринная офтальмопатия [50].

Нарушение функции ЩЖ чаще возникает у женщин, чем у мужчин, — 13 и 3% соответственно [51][52].

Распространенность гипотиреоза, ассоциированного с приемом лития, варьирует в пределах от 6 до 52% по данным различных исследований. Недавний метаанализ, не выявив различий по развитию гипотиреоза между пациентами, получающими литий, и теми, кто получает другие нормотимические средства, подтвердил, что первые имели значительно большую частоту развития литий-ассоциированного зоба (40% против 18%) [53][54][55].

Развитие тиреотоксикоза на фоне приема лития является более редким явлением. В недавнем исследовании показано, что среди пациентов, направляемых в клинику по поводу тиреоидной патологии в течение 12 лет, только 1,4% (23 человека) имели ассоциированный с приемом лития тиреотоксикоз, представленный преимущественно болезнью Грейвса (47,8%). Другими заболеваниями были многоузловой зоб, молчащий и подострый тиреоидит [55].

ТР является редким заболеванием. Распространенность составляет 1,06 случая на 100 000 населения, что соответствует около 0,06% тиреоидэктомий. Среди пациентов чаще заболевают женщины [40].

## 1.4. ОСОБЕННОСТИ КОДИРОВАНИЯ ЗАБОЛЕВАНИЯ ИЛИ СОСТОЯНИЯ (ГРУППЫ ЗАБОЛЕВАНИЙ ИЛИ СОСТОЯНИЙ) ПО МЕЖДУНАРОДНОЙ СТАТИСТИЧЕСКОЙ КЛАССИФИКАЦИИ БОЛЕЗНЕЙ И ПРОБЛЕМ, СВЯЗАННЫХ СО ЗДОРОВЬЕМ

Острый тиреоидит (ОТ):

Подострый тиреоидит (ПТ):

Амиодарониндуцированный тиреоидит:

Цитокининдуцированный тиреоидит:

Тиреопатии, возникшие в результате применения средств, содержащих литий:

Тиреоидит Риделя:

## 1.5. КЛАССИФИКАЦИЯ ЗАБОЛЕВАНИЯ ИЛИ СОСТОЯНИЯ (ГРУППЫ ЗАБОЛЕВАНИЙ ИЛИ СОСТОЯНИЙ)

Существует несколько вариантов классификации тиреоидитов.

I. По гистологической картине.

1.Острые:

2.Подострые:

3.Хронические:

а) аутоиммунные:

б) медикаментозные:

–вследствие применения средств с высоким содержанием йода:

1) амиодарониндуцированные тиреоидиты:

-амиодарониндуцированный гипотиреоз: субклинический/манифестный, транзиторный/постоянный;

-амиодарониндуцированный тиреотоксикоз:

-тип I (йодиндуцированный);

-тип II (деструктивный);

-смешанного типа [6][24];

2) вследствие применения рентгеноконтрастных йодсодержащих веществ [67];

–вследствие цитотоксического действия препаратов:

1) вследствие применения препаратов лития [37];

2) тиреоидиты, вызванные применением некоторых антибактериальных препаратов (Миноциклин, Рифампицин**) [67];

–цитокининдуцированные тиреоидиты:

1) интерфероны, интерлейкин-2;

2) ингибиторы фактора некроза альфа (Инфликсимаб**) [67];

3) моноклональные антитела, ингибирующие контрольные точки иммунного ответа, также известные как чекпойнт-ингибиторы (checkpoint inhibitor): блокирующие цитотоксический Т-лимфоцитассоциированный антиген 4 (CTLA-4);

4) Ипилимумаб**; блокирующие белок запрограммированной клеточной гибели-1 (PD-1) — Пембролизумаб**, Ниволумаб**; блокирующие лиганд рецептора запрограммированной клеточной гибели (PD-L1) — Атезолизумаб**, Дурвалумаб**, Авелумаб**[68];

–вследствие ишемического действия препаратов:

1) ингибиторы протеинкиназы (Сорафениб**) [67];

–фиброзный тиреоидит (зоб Риделя);

–с отсутствием компрессионного синдрома;

–с наличием компрессионного синдрома [69].

II. По функциональному состоянию щитовидной железы.

1.Деструктивные тиреоидиты:

2.Недеструктивные тиреоидиты — другие тиреоидиты, в течении которых нет фазы тиреотоксикоза.

3.Специфические и неспецифические тиреоидиты.

К специфическим относятся тиреоидиты, возникающие при туберкулезе, амилоидозе, саркоидозе, однако данные состояния, с нашей точки зрения, целесообразно осветить в клинических рекомендациях, посвященных соответствующим заболеваниям.

-с компрессионным синдромом;

-без компрессионного синдрома.

## 1.6. КЛИНИЧЕСКАЯ КАРТИНА ЗАБОЛЕВАНИЯ ИЛИ СОСТОЯНИЯ (ГРУППЫ ЗАБОЛЕВАНИЙ ИЛИ СОСТОЯНИЙ)

Симптоматика острого гнойного тиреоидита проявляется клинической триадой:

При остром негнойном тиреоидите основными клиническими жалобами выступают: болевой синдром различной интенсивности, дискомфортные ощущения в области шеи, симптомы тиреотоксикоза [11][41][56][71][72].

Подострый тиреоидит клинически протекает как типичное воспалительное заболевание. В начале развития ПТ пациенты могут иметь продромальные признаки: недомогание, повышение температуры тела до субфебрильных значений, симптомы фарингита, утомляемость. В разгар заболевания ПТ проявляется умеренной или сильной болью в ЩЖ, часто иррадиирующей в уши, челюсть или горло. Боль может начаться очагово и распространяться от одной стороны железы к другой в течение нескольких недель. В редких случаях болезнь может достигать своего пика в течение 3–4 дней и исчезать в течение недели, но, как правило, ПТ характеризуется постепенным развитием [3].

В развитии собственно ПТ выделяют 4 стадии (фазы):

Продромальный период, который обычно предшествует развитию заболевания, может проявляться распространенной миалгией, субфебрильной лихорадкой, общей слабостью, болью в горле (фарингитом). Затем в области ЩЖ появляются боли достаточной интенсивности, иррадиирующие в околоушную область, шею, затылок, иногда отмечается боль при глотании и поворотах головы. ЩЖ обычно несколько увеличена, болезненна при пальпации, часто имеет повышенную плотность. ПТ является распространенной причиной боли в ЩЖ. Отмечаются повышение температуры тела до 38–39 °C, а иногда и до 40 °C, слабость, потливость, раздражительность. В крови выявляется повышенная СОЭ (до 40–60 мм/ч, а иногда и до 100 мм/ч) при чаще неизмененном уровне лейкоцитов и небольшом лимфоцитозе [74].

При ПТ в стадии интенсивных болей на УЗИ выявляются увеличение объема железы, появление в одной или обеих долях зон пониженной эхогенности неправильной формы, без четких контуров. При динамическом наблюдении возможны миграция этих зон, появление их в других участках ЩЖ. Изменения эхограммы сохраняются длительное время и после устранения болевого синдрома и нормализации СОЭ. При исследовании функционального состояния ЩЖ в острой стадии ПТ за счет повышения проницаемости сосудов на фоне воспаления отмечается повышенный выброс ранее синтезированных тиреоидных гормонов и тиреоглобулина. Клинически в этот период заболевания у больных выявляются симптомы тиреотоксикоза, и дифференциальный диагноз проводится с ДТЗ, токсической аденомой и болевой формой АИТ. Больных беспокоят слабость, потливость, раздражительность, беспокойство, нарушение сна, учащенное сердцебиение, тремор рук [57][75][76].

В этот период отмечается выраженное снижение (вплоть до полного отсутствия) захвата радиоактивного йода или 99mТс-пертехнетата ЩЖ — основной дифференциально-диагностический критерий ПТ и ДТЗ, при котором захват радиофармпрепарата будет повышен. В дальнейшем, вследствие нарушения синтеза тиреоидных гормонов и истощения их запасов в ЩЖ, уровни Т3 и Т4 снижаются, и происходит повышение выброса ТТГ. В этот период на сцинтиграммах ЩЖ визуализируется, но захват РФП ниже нормальных значений. В этой стадии ПТ необходимо дифференцировать с АИТ и гипотиреозом. ПТ проходит спонтанно, обычно через несколько месяцев (4–6), иногда он рецидивирует, но стойкий гипотиреоз развивается у меньшей части пациентов (5–25% случаев по разным данным) [4].

Следует отметить, что сразу несколько разновидностей тиреоидита: острый (гнойный), ПТ, некоторые виды лекарственного тиреоидита (наряду с безболевым (молчащим) тиреоидитом, травматическим тиреоидитом, послеродовым тиреоидитом) могут приводить к деструкции ткани ЩЖ, в связи с чем классическим их проявлением служит тиреотоксикоз, чаще всего временный, протекающий как часть классического трехфазного течения (тиреотоксикоз, гипотиреоз, выздоровление). В целом дисфункция ЩЖ, вызванная деструкцией, возникающей вследствие тиреоидита, менее выражена, чем при других формах эндогенного тиреотоксикоза [77].

В клинической картине амиодарониндуцированного гипотиреоза отмечают классические признаки: утомляемость, сухость кожи, зябкость, запоры, сонливость, ухудшение внимания, отечный синдром.

Особенностью клинической картины амиодарониндуцированного тиреотоксикоза является то, что классические симптомы тиреотоксикоза — зоб, потливость, тремор рук, потеря веса — могут быть выражены незначительно или вовсе отсутствовать. В клинической картине, как правило, доминируют сердечно-сосудистые расстройства: учащенное сердцебиение, перебои, одышка при физической нагрузке, утомляемость, рецидивирование нарушений ритма сердца [24][26].

Клиническая картина цитокининдуцированных тиреоидитов весьма вариабельна и зависит от функционального статуса ЩЖ. В 50–70% случаев встречается цитокининдуцированный тиреоидит как деструктивный вариант. Как правило, он характеризуется двухфазным течением: короткая фаза транзиторного тиреотоксикоза сменяется более длительной фазой гипотиреоза, далее возможно восстановление эутиреоидного состояния. Особенно важным с клинической точки зрения является то, что манифестация цитокининдуцированных тиреоидитов возможна на любом этапе лечения (от первых 3 мес — наиболее часто и до отдаленного периода — реже). У подавляющего большинства пациентов с исходно существовавшим АИТ его проявления усугубляются на фоне лечения основного заболевания [50].

По данным метаанализа, у 50% пациентов с АТ-ТПО до терапии возникало нарушение функции ЩЖ по сравнению с 5,4% пациентов исходно без антител [78].

Клиническая картина тиреопатий, возникших в результате применения средств, содержащих литий, вариабельна и зависит от конкретного синдрома.

Тиреоидит Риделя характеризуется увеличением ЩЖ выраженной плотности. Заболевание обычно проявляется обструктивными симптомами, такими как одышка, дисфагия, хрипота из-за поражения структур вокруг ЩЖ. Одышка возникает из-за поражения трахеи, дисфагия связана с вовлечением пищевода, стридорозное дыхание развивается при поражении возвратного гортанного нерва, тромбоз венозного синуса — из-за вовлечения сосудистой сети. При поражении мышц глазного яблока и ретробульбарных тканей возникает вторичный экзофтальм.

При осмотре в передней части шеи пальпируется твердая ЩЖ, которая может не смещаться при глотании из-за спаянности с окружающими тканями. Положительные симптомы Хвостека и Труссо указывают на развитие вторичного гипопаратиреоза [79].

## 2. ДИАГНОСТИКА ЗАБОЛЕВАНИЯ ИЛИ СОСТОЯНИЯ (ГРУППЫ ЗАБОЛЕВАНИЙ ИЛИ СОСТОЯНИЙ), МЕДИЦИНСКИЕ ПОКАЗАНИЯ И ПРОТИВОПОКАЗАНИЯ К ПРИМЕНЕНИЮ МЕТОДОВ ДИАГНОСТИКИ

Диагнозы ОТ, ПТ и ТР основываются на жалобах, данных анамнеза, физикального, лабораторного и инструментального обследования.

Критерии установления диагноза амиодарониндуцированного тиреоидита на основании патогномоничных данных:

1. анамнестических данных о приеме амиодарона**;

2. лабораторных исследований, подтверждающих дисфункцию щитовидной железы (в рамках дифференциальной диагностики);

3. инструментального обследования в рамках дифференциальной диагностики.

Критерии установления диагноза цитокининдуцированных тиреоидитов на основании патогномоничных данных:

1. анамнестических данных о проведении терапии препаратами из группы цитокинового ряда (интерферонами или ингибиторами интерлейкина);

2. лабораторных исследований, подтверждающих дисфункцию щитовидной железы (в рамках дифференциальной диагностики);

3. инструментального обследования в рамках дифференциальной диагностики.

Дифференциальная диагностика острого тиреоидита проводится со следующими заболеваниями:

Наиболее часто проводится дифференциальная диагностика между подострым и острым тиреоидитом, особенно на стадии до образования абсцесса. При ПТ, как правило, отсутствует гипертермия с гектическим характером температуры, болевой синдром присутствует, но интенсивность его значительно меньше, чем при остром тиреоидите. Выраженность симптомов локального воспаления варьирует от незначительных до умеренных.

Может возникнуть необходимость дифференциировать ПТ с IgG4-ассоциированным тиреоидитом, так как изредка при втором заболевании можно обнаружить болезненность в области шеи. К сожалению, клинические диагностические критерии тиреоидита, связанного с IgG4, до конца не сформулированы, условным ориентиром является сохранение болевого синдрома на фоне длительной терапии глюкокортикостероидами, золотым стандартом для диагностики тиреоидита, связанного с IgG4, является иммуноокрашивание IgG4 послеоперационного материала [80][81].

## 2.1. ЖАЛОБЫ И АНАМНЕЗ

Острый тиреоидит

См. раздел «1.6 Клиническая картина».

Подострый тиреоидит

Подробное описание представлено в разделе «1.6. Клиническая картина». Целесообразно также уточнить у пациента сведения о перенесенной вирусной инфекции с целью дифференциальной диагностики ОТ и ПТ.

Амиодарониндуцированный тиреоидит

Уточнить наличие классических жалоб гипотиреоза и тиреотоксикоза, принимая во внимание, что в клинической картине тиреотоксикоза именно кардиальные симптомы, как правило, выступают на первый план.

Важно уточнить прием амиодарона** в анамнезе, даже если терапия была прекращена много месяцев назад. Амиодарониндуцированный гипотиреоз может развиться вскоре после начала приема амиодарона**. Амиодарониндуцированный гипотиреоз развивается раньше, чем амиодарониндуцированный тиреотоксикоз. Для амиодарониндуцированного тиреотоксикоза характерно развитие симптомов в любое время от начала терапии амиодароном** и даже в течение 18 мес после ее отмены [24][26].

Цитокининдуцированный тиреоидит

Клиническая картина вариабельна и зависит от функционального статуса щитовидной железы (гипотиреоза и гипертиреоза).

Тиреоидиты в результате применения средств, содержащих литий

Жалобы аналогичны таковым при нарушениях тиреоидного статуса или структурных изменениях ЩЖ, вызванных другими причинами. В анамнезе следует уточнить прием указанной группы препаратов.

Тиреоидит Риделя

Уровень убедительности рекомендаций С (уровень достоверности доказательств — 5).

Комментарии. Данные симптомы возникают из-за сдавления фиброзной тканью близлежащих органов и тканей; при вовлечении в патологический процесс околощитовидных желез возможно развитие судорог.

## 2.2. ФИЗИКАЛЬНОЕ ОБСЛЕДОВАНИЕ

Острый тиреоидит

См. раздел «1.6. Клиническая картина».

Подострый тиреоидит

См. раздел «1.6. Клиническая картина».

Амиодарониндуцированный тиреоидит

Уровень убедительности рекомендаций C (уровень достоверности доказательств — 5).

Комментарии. Пациентам, получающим терапию амиодароном**, и тем, кому планируется его назначение, необходимо проводить оценку функционального состояния ЩЖ и ее структуры для выявления исходной тиреоидной патологии и мониторинга возможного развития амиодарониндуцированных тиреопатий. Классические признаки тиреоидной патологии могут быть выявлены уже на этапе осмотра. Следует обращать внимание на типичные признаки гипотиреоза (сухая бледная холодная кожа, зябкость, ухудшение внимания, отечный синдром, замедленная речь, слабость, интеллектуальная заторможенность, слабость) и тиреотоксикоза (тремор, необъяснимое снижение веса, миопатия, обострение аритмии или стенокардии, или тепловая непереносимость). В случае амиодарониндуцированного тиреотоксикоза 1 типа возможно наличие эндокринной офтальмопатии.

Наличие узлового зоба при пальпации возможно при амиодарониндуцированном тиреотоксикозе I типа. При амиодарониндуцированном тиреотоксикозе II типа ЩЖ, как правило, мягко-эластичная, не увеличена.

Цитокининдуцированный тиреоидит

Стандартное терапевтическое обследование пациента с прицельной оценкой ЧСС. Стоит уточнить сведения о неожиданном изменении веса (при отсутствии смены привычного режима питания, физической активности и пр.) и возможной непереносимости тепла или холода.

Тиреоидиты в результате применения средств, содержащих литий

Включает в себя пальпацию ЩЖ.

Тиреоидит Риделя

Должно включать в себя пальпацию ЩЖ.

## 2.3. ЛАБОРАТОРНЫЕ ДИАГНОСТИЧЕСКИЕ ИССЛЕДОВАНИЯ

2.3.1. Острый тиреоидит

Уровень убедительности рекомендаций С (уровень достоверности доказательств — 4).

Комментарии. В ОАК выявляется значительное повышение уровня лейкоцитов, за счет нейтрофильного звена лейкопоэза, со сдвигом лейкоцитарной формулы влево. Кроме того, отмечается нарастание СОЭ, результаты которого зависят от выраженности заболевания.

Уровень убедительности рекомендаций С (уровень достоверности доказательств — 5).

Комментарии. У пациентов с ОТ функция ЩЖ нарушается редко. Только при массивном поражении, захватывающем всю долю ЩЖ, могут появляться симптомы тиреотоксикоза деструктивного характера.

2.3.2. Подострый тиреоидит

Уровень убедительности рекомендаций С (уровень достоверности доказательств — 5).

Комментарии. При ПТ отмечается повышение СОЭ >40–60 мм/ч (а в некоторых случаях >100 мм/ч) [4]. При этом уровень лейкоцитов и лейкоцитарная формула чаще в норме, но в редких случаях может встречаться лейкоцитоз.

Уровень убедительности рекомендаций С (уровень достоверности доказательств — 4).

Комментарии. Около 50% пациентов с ПТ имеют начальную тиреотоксическую фазу из-за нерегулируемого высвобождения тиреоидных гормонов из поврежденных фолликулов тиреоцитов (деструктивный характер тиреотоксикоза) [4]. При тиреотоксикозе может выявляться пониженный уровень ТТГ в сочетании с повышенными уровнями св.Т3 и св.Т4. В начальной стадии заболевания уровень ТТГ может быть нормальным. Уровень антител к тиреоглобулину (АТ-ТГ) в сыворотке большинства пациентов может быть повышен в течение нескольких недель после появления симптоматики. Через несколько месяцев антитела исчезают. Однако это обстоятельство не несет дополнительного диагностического значения. Кроме того, приблизительно у 25% пациентов уровень антител может быть нормальным [4, 58, 82].

Уровень убедительности рекомендаций С (уровень достоверности доказательств — 4).

Комментарии. При гипотиреозе, который чаще имеет транзиторный характер, выявляется повышенный уровень ТТГ в сочетании с нормальным — при субклиническом гипотиреозе или сниженным — при манифестном гипотиреозе уровнем св.Т4. В начальной стадии заболевания уровень ТТГ может быть нормальным [4].

2.3.3. Амиодарониндуцированный тиреоидит

Уровень убедительности рекомендаций С (уровень достоверности доказательств — 4).

Комментарии. Предварительное обследование позволяет не только выявить наличие тиреоидной патологии, но и прогнозировать возможное развитие тиреотоксикоза или гипотиреоза после начала терапии, и должно включать исследование крови на ТТГ, св.Т3, св.Т4 (при отклонении ТТГ), АТ-ТПО (присутствие увеличивает риск развития амиодарониндуцированного гипотиреоза во время первого года лечения). Из-за липофильности амиодарона**, позволяющей ему и его производным оставаться в жировой ткани на протяжении месяцев, развитие дисфункции ЩЖ может наступить как во время лечения, так и спустя длительное время после отмены терапии, необходимо исследование ТТГ, св.Т3, св.Т4. Для амиодарониндуцированного гипотиреоза характерно повышение уровня ТТГ (как правило, более 20 МЕ/л), снижение св.Т4. Для амиодарониндуцированного тиреотоксикоза характерно значительное снижение уровня ТТГ, повышение св.Т4, св.Т3.

Уровень убедительности рекомендаций С (уровень достоверности доказательств — 4).

Комментарии. Циркулирующие антитела к АТ-ТГ, АТ-ТПО и рецепторам ТТГ (АТ-рТТГ) в большинстве случаев выявляются у пациентов с амиодарониндуцированным тиреотоксикозом 1 типа, т.е. с исходно имеющейся патологией ЩЖ (преимущественно ДТЗ) [84]. Однако наличие АТ-ТГ и АТ-ТПО в отсутствие АТ-рТТГ не позволяет исключить амиодарониндуцированный тиреотоксикоз 2 типа [85], в связи с чем АТ-рТТГ представляются более надежным маркером, с большей вероятностью указывающим на амиодарониндуцированный тиреотоксикоз 1 типа в случаях, когда он обусловлен латентной болезнью Грейвса, но не токсической адномой или многоузловым токсическим зобом. Таким образом, антитела к ЩЖ (главным образом АТ-рТТГ) свидетельствуют в пользу амиодарониндуцированного тиреотоксикоза 1 типа, но заключение о форме заболевания должно приниматься не изолированно, на основании их наличия/отсутствия, а с учетом результатов других исследований.

2.3.4. Цитокининдуцированный тиреоидит

Уровень убедительности рекомендаций С (уровень достоверности доказательств — 5).

Комментарии. Необходимо исследование ТТГ и АТ (АТ-ТПО и АТ-ТГ) до, во время, а также иногда и после проведения терапии препаратами из группы интерферонов основного заболевания.

Уровень убедительности рекомендаций С (уровень достоверности доказательств — 5).

Комментарии. Если уровень базового ТТГ в пределах референтного лабораторного интервала, а АТ-ТПО и АТ-ТГ отрицательные, то рекомендуется контролировать уровень ТТГ каждые 3 мес до завершения курса терапии.

Если уровень базового ТТГ в пределах референтного лабораторного интервала, но АТ-ТПО и АТ-ТГ положительные, рекомендуется проводить мониторинг уровня ТТГ каждые 2 мес до завершения курса терапии.

2.3.5. Тиреоидиты в результате применения средств, содержащих литий

Уровень убедительности рекомендаций B (уровень достоверности доказательств — 3).

Комментарии. Риск развития тиреоидной патологии возрастает при длительном применении лития. Исследования, необходимые для дифференциальной диагностики между возможными формами литий-ассоциированного тиреоидита: аутоиммунным, безболевым, подострым тиреоидитом, диффузным и узловым токсическим зобом, описаны в соответствующих разделах.

2.3.6. Тиреоидит Риделя

Уровень убедительности рекомендаций С (уровень достоверности доказательств — 3).

Уровень убедительности рекомендаций С (уровень достоверности доказательств — 3).

## 2.4. ИНСТРУМЕНТАЛЬНЫЕ ДИАГНОСТИЧЕСКИЕ ИССЛЕДОВАНИЯ

2.4.1. Острый тиреоидит

Уровень убедительности рекомендаций С (уровень достоверности доказательств — 5).

Комментарии. Ультразвуковое исследование проводится линейным датчиком с частотой 7,5–10 Гц. При начальной стадии заболевания выявляется снижение эхогенности ткани и размытость контуров доли ЩЖ за счет локального отека. В более поздних стадиях выявляются признаки абсцесса — гипоэхогенное образование с жидкостным содержимым. Также УЗИ позволяет оценить реакцию лимфатических узлов не шее.

Уровень убедительности рекомендаций С (уровень достоверности доказательств — 5).

Комментарии. При остром тиреоидите отмечается выраженное снижение накопления радиофармпрепарата (РФП) в ЩЖ, обусловленное нарушением функции ЩЖ в результате воспаления.

Уровень убедительности рекомендаций С (уровень достоверности доказательств — 5).

Комментарии. КТ шеи менее информативно, чем УЗИ, особенно на начальном этапе заболевания. Однако при абсцедировании КТ позволяет диагностировать распространенность процесса, наличие формирующихся или имеющихся свищей. При подозрении на наличие медиастинита и флегмоны шеи является обязательным методом исследования.

2.4.2. Подострый тиреоидит

Уровень убедительности рекомендаций С (уровень достоверности доказательств — 4).

Комментарии. При ПТ при проведении УЗИ можно обнаружить увеличение ЩЖ, диффузную гетерогенность или очаговые «облаковидные» зоны пониженной эхогенности в одной или обеих долях. Описана миграция этих зон. При допплерографии отмечается снижение или нормальный уровень кровотока, в отличие от усиления васкуляризации при болезни Грейвса [4][73][90].

Применение новых технологий, таких как соноэластография, при ПТ способно продемонстрировать заметно сниженную эластичность (повышенную жесткость) при ПТ [91].

Уровень убедительности рекомендаций С (уровень достоверности доказательств — 4).

Комментарии. Отмечается снижение, а иногда и отсутствие захвата РФП во время тиреотоксической фазы ПТ [91].

2.4.3. Амиодарониндуцированный тиреоидит

Уровень убедительности рекомендаций С (уровень достоверности доказательств — 4).

Комментарии. При амиодарониндуцированном тиреотоксикозе I типа наблюдают увеличение объема ЩЖ, наличие одного или нескольких узловых образований; нормальную или повышенную скорость кровотока в ЩЖ. При амиодарониндуцированном тиреотоксикозе II типа узловые образования не визуализируются, скорость кровотока низкая.

Уровень убедительности рекомендаций С (уровень достоверности доказательств — 4).

Комментарии. Традиционная сцинтиграфия ЩЖ с 99mТс-пертехнетатом малоинформативна. Из-за высокого содержания йода в организме ЩЖ не захватывает или очень слабо захватывает 99mTc-пертехнетат (транспорт РФП в тиреоциты обусловлен активностью натрий-йодных симпортеров). В таких условиях определить причину отсутствия захвата (деструкция или блокада йодом) не представляется возможным. В настоящее время появляется больше данных за использование 99mTc-технетрила, который захватывается тиреоцитами путем диффузии и аккумулируется в митохондриях (минуя натрий-йодный симпортер). Отсутствие захвата (или очень слабый захват) 99mTc-технетрила ЩЖ при амиодарониндуцированном тиреотоксикозе II типа обусловлено деструкцией (разрушением клеток). Умеренный или повышенный захват 99mTc-технетрила в ЩЖ может свидетельствовать о наличии смешанной формы или I типа амиодарониндуцированного тиреотоксикоза. Накопление РФП в ЩЖ нельзя однозначно интерпретировать как признак гиперфункции, 99mTc-технетрил имеет тенденцию к повышенному накоплению при различных аутоиммунных заболеваниях ЩЖ (АИТ, узловой зоб, болезнь Грейвса). Ограниченное количество исследований по радионуклидной диагностике амиодарониндуцированных тиреотоксикозов требует дальнейшего изучения темы.

## 2.4.4. Цитокининдуцированный тиреоидит

Уровень убедительности рекомендаций С (уровень достоверности доказательств — 4).

Уровень убедительности рекомендаций С (уровень достоверности доказательств — 5).

Комментарии. При деструктивном тиреоидите будет выявлено снижение или полное отсутствие накопления 99mTc-пертехнетата, а при болезни Грейвса — значительное повышение захвата радиофармпрепарата.

2.4.5. Тиреоидит в результате применения средств, содержащих литий

Уровень убедительности рекомендаций В (уровень достоверности доказательств — 3).

Комментарии. У пациентов, принимающих литий, чаще выявляются диффузное увеличение ЩЖ, а также ее фокальные изменения размерами больше 1 см [99][100].

2.4.6. Тиреоидит Риделя

Уровень убедительности рекомендаций C (уровень достоверности доказательств — 4).

Комментарии. Определяется гипоэхогенная, гиповаскулярная ткань, вовлекающая соседние ткани и иногда сонные артерии; при сцинтиграфии данные образования чаще всего выявляются в виде «холодных» узлов.

Уровень убедительности рекомендаций C (уровень достоверности доказательств — 4).

Комментарии. Ткань имеет серый цвет и каменистую плотность; отсутствует характерное дольчатое строение; выявляется плотное гиалинизированное межклеточное вещество со скудным коллоидом и характерной эозинофильной клеточной инфильтрацией; злокачественные и гигантские клетки отсутствуют; нередко встречается окклюзирующий флебит (окончательный диагноз ТР может быть установлен только на основании данных гистологического исследования послеоперационного материала).

## 2.5. ИНЫЕ ДИАГНОСТИЧЕСКИЕ ИССЛЕДОВАНИЯ

2.5.1. Острый тиреоидит

Уровень убедительности рекомендаций С (уровень достоверности доказательств — 5).

Комментарии. Острый гнойный тиреоидит не требует подтверждения пункционной биопсией. Однако в сложных диагностических случаях с целью дифференциального диагноза с подострым тиреоидитом, анапластической карциномой результаты пункционной биопсии позволяют уточнить диагноз. При подостром тиреоидите выявляются гигантские клетки (полинуклеарные макрофаги), при анапластической карциноме — клетки злокачественной опухоли. Основная роль пункционной биопсии при остром тиреоидите — лечебная. Следует подчеркнуть, что пункционное дренирование применяется только при небольших очагах локального расплавления ЩЖ в результате инфекционного процесса, параллельно с антибактериальной и симптоматической терапией.

2.5.2. Подострый тиреоидит

Уровень убедительности рекомендаций С (уровень достоверности доказательств — 4).

Комментарии. В случае подтверждения ПТ через указанное время должно наступить уменьшение болевого синдрома и постепенное снижение СОЭ [46][47].

Уровень убедительности рекомендаций C (уровень достоверности доказательств — 3).

Комментарии. В отличие от других форм эндогенного тиреотоксикоза при деструктивном тиреотоксикозе уровень общего Т3 может быть в норме.

Уровень убедительности рекомендаций С (уровень достоверности доказательств — 4).

Комментарии. Однако в литературе описана серия клинических случаев сочетания боли в области шеи с повышением СОЭ (разной степени выраженности) в анализе крови с отсутствием эффекта либо непродолжительным эффектом на консервативное лечение, оказавшихся впоследствии анапластическим раком или метастазами рака других локализаций. Данные формы злокачественных новообразований являются чрезмерно агрессивными: промедление на 1–2 нед может существенно повлиять на прогноз пациента. Показания для ТАБ необходимо формулировать на основании данных ультразвуковой картины [105]. При истинном ПТ в цитологическом материале на фоне макрофагов, лейкоцитов, лимфоидных элементов, разрушенных фолликулов отмечается наличие гигантских многоядерных клеток [4][103].

2.5.3. Тиреоидит Риделя

Уровень убедительности рекомендаций C (уровень достоверности доказательств — 4).

Комментарии. Определяется гиподенсная масса, не накапливающая контрастное вещество.

Уровень убедительности рекомендаций С (уровень достоверности доказательств — 3).

Комментарии. Определяется интенсивное поглощение РФП в зоне воспаления; особенно информативно для диагностики очагов фиброза других органов.

## 3. ЛЕЧЕНИЕ, ВКЛЮЧАЯ МЕДИКАМЕНТОЗНУЮ И НЕМЕДИКАМЕНТОЗНУЮ ТЕРАПИИ, ДИЕТОТЕРАПИЮ, ОБЕЗБОЛИВАНИЕ, МЕДИЦИНСКИЕ ПОКАЗАНИЯ И ПРОТИВОПОКАЗАНИЯ К ПРИМЕНЕНИЮ МЕТОДОВ ЛЕЧЕНИЯ

## 3.1. ОСТРЫЙ ТИРЕОИДИТ

Лечение острого гнойного тиреоидита включает в себя обязательное антибактериальное лечение, симптоматическую терапию, пункционное дренирование под контролем УЗИ и хирургическое лечение.

3.1.1. Консервативное лечение

Уровень убедительности рекомендаций С (уровень достоверности доказательств — 5).

Комментарии. Рекомендация обусловлена выраженностью клинической картины и быстрой скоростью развития заболевания.

Уровень убедительности рекомендаций С (уровень достоверности доказательств — 5).

Комментарии. Начальная стадия заболевания успешно лечится без применения хирургического лечения. Однако следует отметить редкость обращения пациентов на начальном периоде развития ОТ. Основным методом консервативного лечения является антибиотикотерапия препаратами широкого спектра (цефалоспорины; бета-лактамные антибактериальные препараты: пенициллины; макролиды). Также на этом этапе применяется противовоспалительное лечение нестероидными противовоспалительными препаратами (НПВП). В качестве симптоматического лечения применяются препараты группы НПВП, а при развитии деструктивного тиреотоксикоза — бета-адреноблокаторы. При кровоизлияниях с целью декомпрессии иногда применяется пункционное дренирование под контролем УЗИ в асептических условиях.

3.1.2. Хирургическое лечение

Уровень убедительности рекомендаций С (уровень достоверности доказательств — 5).

Комментарии. При начале лизиса участков ткани ЩЖ параллельно с антибактериальной терапией эффективно использовать пункционное дренирование. Метод используется только при малых очагах поражения, не более 1,0–1,5 см в диаметре. Под контролем УЗИ методом «free-hand» проводится пункция, дренирование гнойного очага иглой 21G. Любые сомнения в эффективности пункционного дренирования (персистирование абсцесса после 2 пункционных дренирований) должны быть решены в пользу хирургического вмешательства (гемитиреоидэктомии). Параллельно с пункционным дренированием используются оговоренная выше антибактериальная терапия и симптоматическое лечение.

Уровень убедительности рекомендаций С (уровень достоверности доказательств — 4).

Комментарии. В подавляющем большинстве случаев объем оперативного вмешательства — гемитиреоидэктомия. Хирургическое лечение проводится в экстренном порядке, под общим обезболиванием, позволяющим выполнить адекватную санацию очага поражения. Гемитиреоидэктомия выполняется типичным образом, по возможности, не вскрыв полость абсцесса с наименьшей травматизацией окружающих тканей и фасциальных пространств. Рана обязательно промывается растворами антисептиков (гидроксиметилхиноксалиндиоксид, хлоргексидин**), устанавливается сторожевой дренаж, который выводится через контрапертурное отверстие. Рана на шее ушивается. В случае осложнений, таких как медиастинит, оперативное лечение дополняется санацией и дренированием средостения. При свищах ЩЖ с трахеей дефект ушивается после экономного иссечения пораженных тканей. При наличии фистулы левой доли щитовидной железы с грушевидным синусом в детском возрасте обязательными являются иссечение фистулы и ушивание грушевидного синуса [111][112].

Уровень убедительности рекомендаций С (уровень достоверности доказательств — 5).

Комментарии. Ткани шеи, окружающие абсцедированную долю ЩЖ, крайне инфильтрированы. В такой ситуации поиск и идентификация возвратно-гортанного нерва со стороны поражения могут быть осложнены воспалительной инфильтрацией. Использование постоянного нейромониторинга позволяет снизить вероятность пареза возвратно-гортанного нерва [113][114].

Как правило, после операции продолжается антибактериальная терапия, особенно в случаях осложненного течения. Также используются препараты симптоматического лечения и инфузионно-дезинтоксикационная терапия.

## 3.2. ПОДОСТРЫЙ ТИРЕОИДИТ

Лечение ПТ в подавляющем большинстве случаев консервативное.

3.2.1. Консервативное лечение

Классическими препаратами для лечения ПТ являются НПВП и глюкокортикостероиды (ГКС) [115]. В качестве симптоматических средств при необходимости используют бета-адреноблокаторы в тиреотоксическую фазу.

Уровень убедительности рекомендаций С (уровень достоверности доказательств — 4).

Комментарии. Бета-адреноблокаторы рекомендуются, по мере необходимости, пациентам с симптоматическим тиреотоксикозом, особенно пожилым и пациентам с ЧСС>90 в минуту в состоянии покоя, а также лицам с сердечно-сосудистыми заболеваниями (за исключением противопоказаний; с осторожностью при бронхиальной астме). Доза индивидуальна, чаще всего используется 40–120 мг/сут пропранолола** или 25–50 мг/сут атенолола** [10, 116, 118, 119]. НПВП обеспечивают облегчение боли у пациентов с легкими симптомами и должны рассматриваться как терапия первой линии. При применении НПВП медиана времени разрешения боли составляет 5 нед (возможный диапазон 1–20 нед). Исторически в качестве НПВП применялась ацетилсалициловая кислота**, однако имеются данные о ее способности вытеснять Т4 из связи с белком, в связи с чем предпочтительно использовать другие препараты. Возможно применение ибупрофена**, но наиболее предпочтительными являются препараты пролонгированного действия: напроксен. Режим применения и дозы напроксена: 500–1000 мг/сут в 2 приема (утром и вечером) во время еды, с возможным переходом в режим поддерживающей дозы — 500 мг/сут в 1 или 2 приема [4][119].

Уровень убедительности рекомендаций С (уровень достоверности доказательств — 4).

Комментарии. Пациентам, у которых отсутствует ответ на лечение полными дозами НПВП в течение нескольких дней, должны быть назначены ГКС. Стандартные рекомендации заключаются в использовании преднизолона** в дозах 20–30 мг/сут, причем критериями снижения дозы ГКС служат уменьшение или исчезновение болей в ЩЖ (через 24–72 ч), нормализация СОЭ (контроль должен быть осуществлен через 2 нед от начала лечения). Отсутствие клинического эффекта от применения ГКС в течение 2 нед может быть диагностическим признаком иного характера патологического процесса в ЩЖ. Однако при выраженной тяжести симптомов возможно также назначение 40 мг ежедневно в течение 1–2 недель с последующим постепенным снижением дозы в течение 2–4 нед или дольше, в зависимости от клинического ответа [4][120][121].

Результаты одного из исследований продемонстрировали, что более низкая начальная суточная доза: 15 мг преднизолона** с уменьшением на 5 мг каждые 2 нед была эффективной. Однако 20% пациентов потребовалось более 8 нед, чтобы прекратить прием ГКС [117]. Данный вид терапии можно рекомендовать в качестве альтернативной схемы в особых случаях.

На фоне лечения ГКС отмечаются уменьшение объема ЩЖ, положительная эхографическая динамика. Нормализация эхографической картины ЩЖ у больных запаздывает по сравнению с нормализацией клинико-лабораторных данных.

Уровень убедительности рекомендаций С (уровень достоверности доказательств — 5).

Комментарии. Рекомендация обусловлена тем, что характер тиреотоксикоза при ПТ — деструктивный, назначение антитиреоидных препаратов не обосновано.

Уровень убедительности рекомендаций С (уровень достоверности доказательств — 4).

Комментарии. Назначение антибиотикотерапии неэффективно, так как этиология заболевания, предположительно, вирусная.

Уровень убедительности рекомендаций А (уровень достоверности доказательств — 4).

Комментарии. Левотироксин натрия** может быть использован во время гипотиреоидной стадии, но должен быть отменен через 3–6 мес, когда в типичном случае наступает восстановление нормальной функции ЩЖ, что подтверждается лабораторными тестами. Доза подбирается индивидуально, в зависимости от выраженности гипотиреоза.

3.2.2. Хирургическое лечение

В литературе описаны единичные случаи ПТ, устойчивого к длительному лечению высокими дозами преднизолона** (50 мг/сут и выше), при этом авторы рассматривают возможность тиреоидэктомии [124]. Однако утверждение является спорным, кроме того, в данном случае особое значение приобретает тщательная дифференциальная предоперационная (с целью решения вопроса о целесообразности и объеме радикального лечения в случае его необходимости) диагностика.

## 3.3. АМИОДАРОНИНДУЦИРОВАННЫЙ ТИРЕОИДИТ

3.3.1. Консервативное лечение

Уровень убедительности рекомендаций В (уровень достоверности доказательств — 4).

Комментарии. При прекращении терапии амиодароном** может наступить спонтанная ремиссия гипотиреоза, однако вероятность этого при наличии индуцированного (или исходного) аутоиммунного заболевания невелика [125]. С учетом того, что развитие гипотиреоза без существенных трудностей компенсируется приемом левотироксина натрия**, мы рекомендуем не прекращать терапию амиодароном**, которая во многих случаях является жизненно необходимой. Критерии эффективности лечения левотироксином натрия**: поддержание уровня ТТГ в пределах референсного диапазона ближе к его верхней границе или немного ее превышающего и уровня св.Т4 и св.Т3 — в пределах референса. Дозы левотироксина натрия** могут быть выше обычных, так как амиодарон** — ингибитор конверсии Т4 в Т3. Поскольку пациенты, получающие амиодарон**, — это больные с тяжелыми кардиальными заболеваниями, терапию левотироксином натрия** начинают с небольших доз (12,5–25 мкг утром натощак) и увеличивают дозу с интервалом в 4–6 нед. В дальнейшем мониторирование содержания ТТГ необходимо проводить 1 раз в 3 мес [24][126].

Уровень убедительности рекомендаций В (уровень достоверности доказательств — 4).

Комментарии. При выявлении субклинического гипотиреоза возможно продолжение приема амиодарона** без назначения терапии левотироксином натрия** ввиду возможного ухудшения состояния сердечно-сосудистой системы (особенно у пожилых пациентов) [127]. Субклинический гипотиреоз не обязательно переходит в манифестный [128], но, с учетом риска прогрессирования, следует регулярно контролировать функцию ЩЖ.

Уровень убедительности рекомендаций С (уровень достоверности доказательств — 4).

Комментарии. Вопрос о возможности продолжать лечение амиодароном** при развитии амиодарониндуцированного тиреоидита дискутабелен, поскольку: нередко контроль аритмии без него невозможен, липофильность обуславливает отсутствие улучшения после отмены, т.к. препарат остается в организме в течение месяцев после отмены, а кроме того, амиодарон** ингибирует конверсию Т4 в Т3, в том числе и в сердечной ткани, в связи с чем его отмена может привести к усилению тиреотоксикоза. Описаны случаи смерти после прекращения терапии амиодароном** у пациентов с амиодарониндуцированным тиреотоксикозом. С учетом того, что II тип является самолимитирующимся заболеванием, отмена амиодарона** может привести к более скорому улучшению, однако такие пациенты могут эффективно отвечать на терапию ГКС в любом случае. Отдельно обсуждается вопрос о возможности возобновления терапии амиодароном** после купирования заболевания. После ликвидации амиодарониндуцированного тиреотоксикоза 2 типа прием амиодарона** может быть возобновлен. Показано, что рецидив амиодарониндуцированного тиреотоксикоза 1 типа возникает в 70% случаев, если не проводилась превентивная терапия антитиреоидными препаратами или РЙТ, в связи с чем следует выполнить радикальное лечение перед возобновлением приема амиодарона**. Для II типа таких данных о рецидиве нет, и возобновление приема амиодарона** возможно.

Уровень убедительности рекомендаций В (уровень достоверности доказательств — 4).

Комментарии. Для подавления синтеза тиреоидных гормонов при амиодарониндуцированном тиреотоксикозе 1 типа рекомендуется применение антитиреоидных препаратов. Из-за сниженной эффективности их воздействия на тиреоидную ткань с высоким содержанием йода требуются более высокие дозировки (#тиамазол** — 40–60 мг/сут, #пропилтиоурацил — 600–800 мг/сут), а сроки медикаментозной компенсации удлиняются. Эутиреоз, как правило, восстанавливается через 6–12 нед. Доза антитиреоидного препарата должна снижаться после лабораторной компенсации тиреотоксикоза (нормализация уровней св.Т4 и св.Т3) [24][94][126][136].

Уровень убедительности рекомендаций A (уровень достоверности доказательств — 2).

Комментарии. При легком течении тиреотоксикоза возможно динамическое наблюдение. При тяжелом течении назначают ГКС (преднизолон** 20–80 мг в день) в течение 7–12 нед. Отмена ГКС в более ранние сроки (через 2–3 нед) ведет к рецидиву тиреотоксикоза.

Уровень убедительности рекомендаций С (уровень достоверности доказательств — 5).

Комментарии. Будучи классифицированными как имеющие амиодарониндуцированный тиреотоксикоз типа I или типа II, пациенты часто не реагируют на терапию, специально направленную на этот подтип, что обусловлено как трудностями в дифференциальной диагностике, так и наличием форм со смешанным патогенезом. С учетом опасности тиреотоксикоза и необходимости скорейшего его купирования у пациентов с тяжелой кардиальной патологией оправданным является назначение комбинированной терапии.

3.3.2. Хирургическое лечение

Уровень убедительности рекомендаций С (уровень достоверности доказательств — 4).

Комментарии. Пациентам с тиреотоксикозом при амиодарониндуцированном тиреоидите, не отвечающим на медикаментозную терапию, должна быть предложена тиреоидэктомия, прежде чем разовьются тяжелые кардиальные осложнения из-за неадекватно контролируемого тиреотоксикоза. Следует объяснить, что, хотя тиреоидэктомия в этой ситуации относится к числу операций высокого риска, запаздывание с ее проведением ведет к еще большему риску смерти. Вид анестезии при тиреоидэктомии в данном случае допускает местную анестезию, что может быть предпочтительным для тяжелых пациентов. В настоящее время опубликовано несколько хирургических серий с участием пациентов с амиодарониндуцированными тиреоидитами, которые в целом дали благоприятные результаты.

3.3.3. Иное лечение

Уровень убедительности рекомендаций В (уровень достоверности доказательств — 3).

Комментарии. В настоящее время данные об опыте применения РЙТ у пациентов с амиодарониндуцированным тиреотоксикозом очень ограничены. Если исходить из патогенетических особенностей заболевания, можно предполагать, что при I типе с высоким захватом РФП эффективность должна быть сопоставимой с таковой при классической болезни Грейвса и функциональной автономии, а при II типе и продолжении приема амиодарона** — низкой вследствие йодной нагрузки и деструктивного процесса, успешного применения РЙТ для предотвращения рецидива у эутиреоидных пациентов с эпизодом амиодарониндуцированного тиреотоксикоза в анамнезе, которым было запланировано возобновление приема амиодарона**. Кроме того, описаны случаи, демонстрирующие успешность достижения гипо- или эутиреоза после одно- или двукратного курса РЙТ, в том числе и у пациентов в состоянии тиреотоксикоза, с низким захватом РФП и непрерывно продолжавших прием амиодарона**. С учетом безопасности применения РЙТ, отсутствия побочных эффектов и хорошей переносимости, следует рассматривать ее в качестве радикального метода лечения, как альтернативу тиреоидэктомии при неэффективности консервативного лечения. Использование стимуляции тиротропином альфа в рамках подготовки таких пациентов к РЙТ не рекомендовано, т.к. это может привести к повышению уровня тиреоидных гормонов и спровоцировать ухудшение состояния. Схема ведения пациентов с амиодарониндуцированными тиреопатиями представлена в приложении Б.

## 3.4. ЦИТОКИНИНДУЦИРОВАННЫЕ ТИРЕОИДИТЫ

3.4.1. Консервативное лечение

Комментарии. Учитывая, что классический вариант цитокининдуцированных тиреоидитов представлен деструктивным тиреоидитом, схема ведения пациентов при цитокининдуцированном тиреоидите представлена на рисунке 1.

**Figure:**
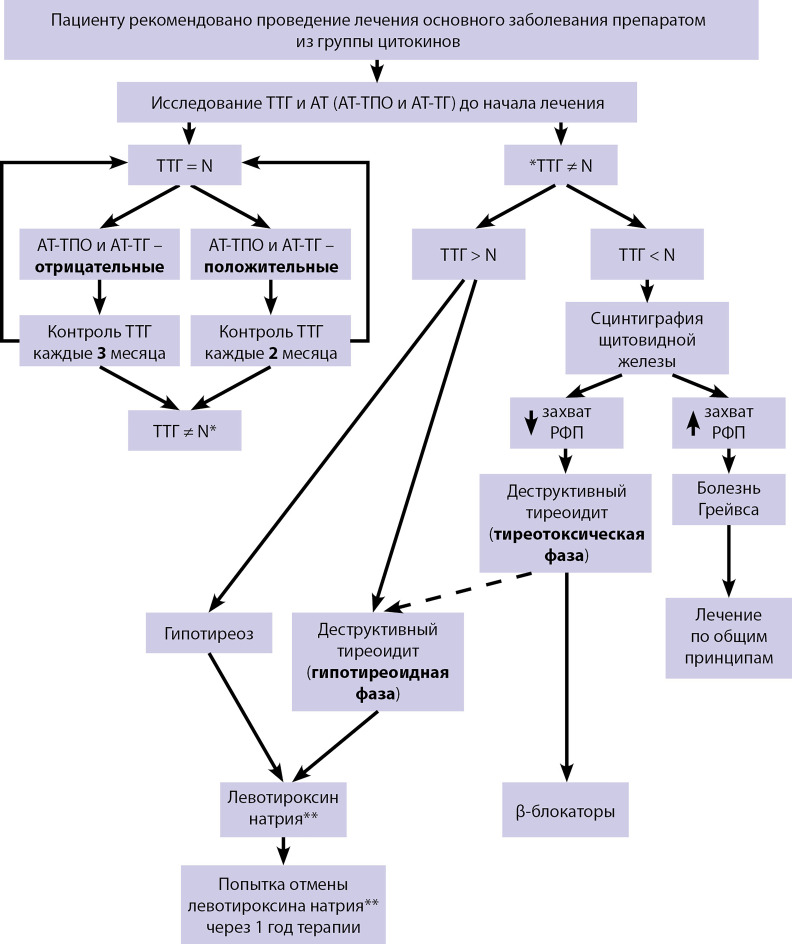
Рисунок 1. Схема ведения пациентов при цитокининдуцированном

Комментарии. Цитокининдуцированный тиреотоксикоз наиболее часто является транзиторным и самостоятельно купируется. В качестве симптоматической терапии могут быть использованы бета-адреноблокаторы. Антитиреоидные препараты могут вызвать гепатотоксический эффект, что может усугубить основное заболевание, по поводу которого проводится терапия препаратами из группы интерферонов, и патогенетически не обоснованы.

Уровень убедительности рекомендаций С (уровень достоверности доказательств — 5).

Комментарии. Поскольку у большинства пациентов гипотиреоз является транзиторным, через год делается попытка отмены левотироксина натрия**.

При развитии у пациента болезни Грейвса лечение проводится по общим принципам лечения данного заболевания.

Схема ведения пациентов с цитокининдуцированным тиреоидитом представлена в приложении Б.

## 3.5. ТИРЕОИДИТЫ, ВОЗНИКШИЕ В РЕЗУЛЬТАТЕ ПРИМЕНЕНИЯ СРЕДСТВ, СОДЕРЖАЩИХ ЛИТИЙ

3.5.1. Консервативное лечение

Уровень убедительности рекомендаций С (уровень достоверности доказательств — 4).

Комментарии. Большинство пациентов с литий-ассоциированным гипотиреозом восстанавливают эутиреоидный статус после прекращения терапии. Тем не менее препараты лития являются основным компонентом в комплексном лечении биполярного расстройства. Отказ от нее из-за развившейся тиреоидной патологии, которая удовлетворительно компенсируется медикаментозно и нередко имеет транзиторный характер, не обоснован [149][150].

Тактика ведения тиреопатий, ассоциированных с приемом лития, не имеет принципиальных отличий и зависит от этиопатогенетического варианта заболевания. Заместительная терапия левотироксином натрия** показана при гипотиреозе. При развитии безболевого тиреоидита рекомендовано лечение бета-адреноблокаторами для купирования симптомов. В некоторых случаях следует рассматривать применение ГКС, но только при выраженном тиреотоксикозе. Применять ГКС следует с осторожностью с учетом их возможного негативного влияния на психическое состояние (развитие маниакальных эпизодов у пациентов с биполярным расстройством). Тактика при болезни Грейвса и узловом токсическом зобе описана в соответствующих рекомендациях [149][150][151].

## 3.6. ТИРЕОИДИТ РИДЕЛЯ

При ТР не существует единого мнения в выборе как консервативной терапии, так и оптимального объема операции ввиду отсутствия результатов исследований, что связано с редкостью заболевания.

Лечение ТР включает в себя:

Кроме того, при развитии гипопаратиреоза применяются препараты кальция и колекальциферол [106] (описаны в соответствующих Клинических рекомендациях).

3.6.1. Консервативное лечение

Уровень убедительности рекомендаций С (уровень достоверности доказательств — 4).

Комментарии. ГКС рассматривают как основу консервативной терапии, противовоспалительное действие которых наиболее эффективно при использовании на ранних стадиях заболевания. Эффективной дозы в настоящее время не существует.

Уровень убедительности рекомендаций С (уровень достоверности доказательств — 4).

Комментарии. Тамоксифен** представляет собой селективный модулятор рецептора эстрогена (SERM), используемый для лечения ТР и других проявлений системного фиброза. Он индуцирует фактор роста опухоли бета (TGF-β), который является мощным ингибитором роста фиброзной ткани.

3.6.2. Хирургическое лечение

Уровень убедительности рекомендаций С (уровень достоверности доказательств — 5).

Комментарии. Существует предположение о возможной так называемой «стадийности» ТР, поэтому вероятность ремиссии, в том числе при минимальном оперативном вмешательстве, вероятно, будет зависеть от того, на какой стадии проведено оперативное вмешательство. Убедительных четких данных нет.

Схема ведения пациентов при тиреоидите Риделя представлена на рисунке 2.

**Figure:**
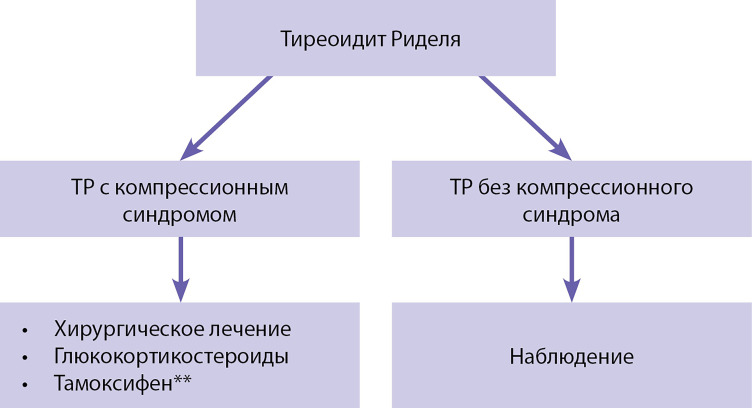
Рисунок 2. Схема ведения пациентов при тиреоидите

## 4. МЕДИЦИНСКАЯ РЕАБИЛИТАЦИЯ, МЕДИЦИНСКИЕ ПОКАЗАНИЯ И ПРОТИВОПОКАЗАНИЯ К ПРИМЕНЕНИЮ МЕТОДОВ РЕАБИЛИТАЦИИ

Не разработана.

## 5. ПРОФИЛАКТИКА И ДИСПАНСЕРНОЕ НАБЛЮДЕНИЕ, МЕДИЦИНСКИЕ ПОКАЗАНИЯ И ПРОТИВОПОКАЗАНИЯ К ПРИМЕНЕНИЮ МЕТОДОВ ПРОФИЛАКТИКИ

Профилактикой ОТ является своевременное лечение первичных инфекций.

Специфическая профилактика ПТ не разработана. Диспансерное наблюдение зависит от тяжести состояния пациента и длительности определенной фазы заболевания.

Специфическая профилактика амиодарониндуцированных тиреоидитов не разработана.

Специфическая профилактика цитокининдуцированных тиреоидитов не разработана.

Диспансерное наблюдение: зависит от тяжести состояния пациента (прежде всего, основного заболевания, в лечении которого используются препараты из группы цитокинового ряда), а также от клинической картины при развитии тиреоидита. Частота, объем контрольных исследований и консультации специалистов определяются индивидуально.

Специфическая профилактика тиреопатий, возникших в результате применения средств, содержащих литий, не разработана.

Профилактики ТР в настоящее время нет, однако следует обратить внимание на провоцирующие факторы и условия, усугубляющие симптомы обструкции, такие как курение, инфекционные заболевания верхних дыхательных путей, загрязненность окружающего воздуха промышленными и другими отходами, специфика некоторых профессий, климатические условия. Диспансерное наблюдение зависит от тяжести состояния пациента и вовлечения в патологический процесс окружающих органов и тканей, частота контрольных исследований и консультации специалистов определяюется индивидуально.

## 6.ОРГАНИЗАЦИЯ ОКАЗАНИЯ МЕДИЦИНСКОЙ ПОМОЩИ

## 6.1. ОСТРЫЙ ТИРЕОИДИТ

Показания для госпитализации (экстренной) в медицинскую организацию (стационар) при остром тиреоидите — во всех случаях.

Показания к выписке:

## 6.2. ПОДОСТРЫЙ ТИРЕОИДИТ

Показания для госпитализации (экстренной) в медицинскую организацию (стационар) при ПТ имеются при наличии у пациента:

Показания к выписке пациента из медицинской организации при ПТ:

1. купирование болевого синдрома;

2. устранение выраженных симптомов тиреотоксикоза.

## 6.3. АМИОДАРОНИНДУЦИРОВАННЫЙ ТИРЕОИДИТ

Показания для госпитализации пациентов с амиодарониндуцированным тиреоидитом в медицинскую организацию:

Показания к выписке пациента с амиодарониндуцированным тиреоидитом из медицинской организации:

## 6.4. ЦИТОКИНИНДУЦИРОВАННЫЙ ТИРЕОИДИТ

Показания для госпитализации (экстренной) в медицинскую организацию (стационар):

Показания для госпитализации (плановой) в медицинскую организацию (стационар):

Показания к выписке из медицинской организации (стационара):

## 6.5. ТИРЕОПАТИИ, ВОЗНИКШИЕ В РЕЗУЛЬТАТЕ ПРИМЕНЕНИЯ СРЕДСТВ, СОДЕРЖАЩИХ ЛИТИЙ

Показания для госпитализации (экстренной) в медицинскую организацию (стационар):

## 6.6. ТИРЕОИДИТ РИДЕЛЯ

Показания для плановой госпитализации:

Показания для экстренной госпитализации:

Показания к выписке пациента из стационара:

Общие для всех заболеваний.

## 7. ДОПОЛНИТЕЛЬНАЯ ИНФОРМАЦИЯ (В ТОМ ЧИСЛЕ ФАКТОРЫ, ВЛИЯЮЩИЕ НА ИСХОД ЗАБОЛЕВАНИЯ ИЛИ СОСТОЯНИЯ)

## 7.1. ОСТРЫЙ ТИРЕОИДИТ

Осложнениями острого гнойного тиреоидита являются патологии распространения инфекции в результате несвоевременного лечения. К таким заболеваниям относятся:

Прогноз при ОТ в случае своевременного начала лечения благоприятен. Рецидивирующее течение чаще всего выявляется в детском возрасте, редко у взрослых. Прогноз при осложненном течении зависит от запущенности заболевания. Смертность при этом достигает 12% [14].

## 7.2. ПОДОСТРЫЙ ТИРЕОИДИТ

Наличие или отсутствие АИТ не имеет дополнительного значения при ПТ [4].

При рецидиве заболевания рекомендовано повторное проведение диагностики и возобновление лечения.

В целом прогноз достаточно благоприятный: приблизительно у 90% пациентов наблюдаются полное и спонтанное выздоровление и восстановление нормальной функции ЩЖ. Однако морфологически у таких пациентов в ткани ЩЖ может образовываться рубцовая ткань между островками остаточной паренхимы, хотя какие-либо симптомы отсутствуют. ПТ может рецидивировать в 2,8–4% случаев [4]. Чуть менее чем у 10% пациентов может развиваться стойкий гипотиреоз, что требует постоянной заместительной терапии левотироксином натрия**, при этом наличие двусторонних гипоэхогенных участков на УЗИ ЩЖ во время установления диагноза представляется ценным прогностическим маркером в отношении развития стойкого гипотиреоза в дальнейшем [152]. При анализе литературы встречаются противоречивые данные, однако большинство исследователей сходятся во мнении, что способ консервативного лечения (НПВП или ГКС) преимущественно не влияет на прогноз в отношении развития стойкого гипотиреоза [58, 153].

Уровни СОЭ и С-реактивного белка на момент постановки диагноза не влияют на рецидив или развитие постоянного гипотиреоза [129].

## 7.3. АМИОДАРОНИНДУЦИРОВАННЫЙ ТИРЕОИДИТ

**Table:** Таблица 1. Отличительные особенности амиодарониндуцированного тиреотоксикоза (АМИТ) I и II типа

Признак	АМИТ I	АМИТ II
Этиология и патогенез
Дефицит йода в регионе	Да	Нет
Длительность приема амиодарона**	Менее 1–2 лет	Более 1–2 лет
Исходная патология ЩЖ	Есть	Нет
Патогенетический механизм	Увеличение синтеза тиреоидных гормонов ЩЖ под воздействием йода вследствие запуска аутоиммунного процесса, образования АТ-рТТГ и формирования йодиндуцированной болезни Грейвса или индукции функциональной автономии	Чрезмерное высвобождение тиреоидных гормонов вследствие деструкции ткани ЩЖ из-за цитотоксического воздействия амиодарона**
Диагностика
Пальпация ЩЖ	Узловой или диффузный зоб	Норма или небольшой зоб, чувствительный при пальпации
АТ-рТТГ	Определяются при ДТЗ, но могут не выявляться при токсической аденоме или многоузловом токсическом зобе	Отсутствуют
св.Т3 и св.Т4	Выраженное повышение уровней св.Т4 и св.Т3	Преимущественное повышение св.Т4
УЗИ ЩЖ	Узловой или диффузный зоб	Норма или малых размеров, гипоэхогенная
Цветовое допплеровское картирование при УЗИ ЩЖ	Выраженная васкуляризация	Отсутствие васкуляризации
Захват РФП в ходе сцинтиграфии ЩЖ с 99mTc-технетрилом	Норма или повышен	Снижен или отсутствует
Лечение
Самолимитирующееся заболевание	Нет	Да
Терапия	Антитиреоидные препараты	Глюкокортикостероиды
Гипотиреоз в исходе консервативного лечения	Нет	Да

Выделены наиболее значимые диагностические критерии.

## 7.4. ЦИТОКИНИНДУЦИРОВАННЫЙ ТИРЕОИДИТ

Факторы риска развития цитокининдуцированного тиреоидита: женский пол, наличие вируса гепатита С, АТ-ТПО и к ТГ.

Прогноз определяется наличием у пациента сопутствующей тяжелой соматической патологии или прогрессированием основного заболевания (в лечении которого используются препараты из группы цитокинов).

## 7.5. ТИРЕОИДИТ РИДЕЛЯ

При ТР прогноз во многом определяет наличие у пациента сопутствующей тяжелой соматической патологии.

## КРИТЕРИИ ОЦЕНКИ КАЧЕСТВА МЕДИЦИНСКОЙ ПОМОЩИ

Острый тиреоидит

**Table:** 

1.	Выполнен ОАК с оценкой лейкоцитарной формулы и СОЭ	С	4
2.	Выполнено исследование уровня тиреотропного гормона (ТТГ) в крови при наличии клинической картины тиреотоксикоза	С	5
3.	Выполнено УЗИ ЩЖ	С	5
4.	Назначена антибактериальная терапия	С	5
5.	Выполнено пункционное дренирование в сочетании с антибактериальной терапией при малых очагах поражения или гемитиреоидэктомия при абсцедировании	С	5

Подострый тиреоидит

**Table:** 

1.	Выполнен ОАК с оценкой СОЭ	С	5
2.	Выполнено исследование уровня тиреотропного гормона (ТТГ), свободного трийодтиронина (св.Т3), свободного тироксина (св.Т4) в крови в тиреотоксическую фазу	С	4
3.	Выполнено исследование уровня тиреотропного гормона (ТТГ), свободного тироксина (св.Т4) в крови в гипотиреоидную фазу	С	4
4.	Проведено УЗИ ЩЖ	С	4
5.	Назначены бета-адреноблокаторы, НПВП или ГКС при необходимости в указанных дозах	С	4

Медикаментозные тиреоидиты — заболевания ЩЖ, возникшие в результате применения лекарственных средств.

Амиодарониндуцированный тиреоидитЦитокининдуцированные тиреоидиты

**Table:** 

№	Критерии качества	Уровень убедительности рекомендаций	Уровень достоверности доказательств
1.	Выполнено исследование уровня ТТГ и определение содержания АТ-ТПО и АТ-ТГ в сыворотке крови при проведении лечения интерферонами или ингибиторами интерлейкина	С	5
2.	Выполнено динамичное исследование уровня ТТГ в крови (каждые 2–3 мес) при проведении лечения интерферонами или ингибиторами интерлейкина	С	5
3.	Выполнено УЗИ ЩЖ при развитии дисфункции ЩЖ на фоне лечения препаратами интерферонов или интерлейкинов.	С	5
4.	Выполнена сцинтиграфия ЩЖ с 99mTc-пертехнетатом при формировании тиреотоксикоза на фоне лечения интерферонами или ингибиторами интерлейкина	С	5
5.	Проведено лечение нарушения функции ЩЖ, развившегося на фоне проведения лечения интерферонами или ингибиторами интерлейкина, в зависимости от фазы заболевания.	С	5

Тиреоидит вследствие приема средств, содержащих литий

**Table:** 

1.	Выполнено исследование уровня ТТГ в крови и определение содержания АТ-ТПО перед назначением терапии препаратами лития. Контроль ТТГ с интервалом 6–12 мес на фоне лечения (1 раз в 3 мес при наличии положительного титра АТ-ТПО)	B	3
2.	Выполнено УЗИ ЩЖ у пациента, получающего терапию препаратами лития, — исходно	В	3

Тиреоидит Риделя

**Table:** 

1.	Выполнено исследование уровня ТТГ крови	2	А
2.	Выполнено УЗИ ЩЖ	4	С
3.	Выполнено КТ органов шеи с контрастным усилением — при необходимости	4	С
4.	Проведено лечение в полном объеме: консервативное или хирургическое (при наличии синдрома компрессии)	5	С
5.	В случае хирургического лечения выполнено макроскопическое и гистологическое исследование послеоперационного материала	4	С
